# Porphyrin-Based Supramolecular Self-Assemblies: Construction, Charge Separation and Transfer, Stability, and Application in Photocatalysis

**DOI:** 10.3390/molecules29246063

**Published:** 2024-12-23

**Authors:** Yingxu Hu, Jingfeng Peng, Rui Liu, Jing Gao, Guancheng Hua, Xiangjiang Fan, Shengjie Wang

**Affiliations:** College of Chemistry and Chemical Engineering, China University of Petroleum, Qingdao 266580, China

**Keywords:** porphyrin, supramolecular photocatalyst, mechanism for charge separation, intrinsic relationship between the supramolecular structure and property, application

## Abstract

As a key means to solve energy and environmental problems, photocatalytic technology has made remarkable progress in recent years. Organic semiconductor materials offer structural diversity and tunable energy levels and thus attracted great attention. Among them, porphyrin and its derivatives show great potential in photocatalytic reactions and light therapy due to their unique large-π conjugation structure, high apparent quantum efficiency, tailorable functionality, and excellent biocompatibility. Compared to unassembled porphyrin molecules, supramolecular porphyrin assemblies facilitate the solar light absorption and improve the charge transfer and thus exhibit enhanced photocatalytic performance. Herein, the research progress of porphyrin-based supramolecular assemblies, including the construction, the regulation of charge separation and transfer, stability, and application in photocatalysis, was systematically reviewed. The construction strategy of porphyrin supramolecules, the mechanism of charge separation, and the intrinsic relationship of assembling structure-charge transfer-photocatalytic performance received special attention. Surfactants, peptide molecules, polymers, and metal ions were introduced to improve the stability of the porphyrin assemblies. Donor-acceptor structure and co-catalysts were incorporated to inhibit the recombination of the photoinduced charges. These increase the understanding of the porphyrin supramolecules and provide ideas for the design of high-performance porphyrin-based photocatalysts.

## 1. Introduction

Widespread utilization of fossil energy, including coal, oil, and natural gas, results in serious energy shortages and environmental pollution accompanied by rapid development and industrialization [1,2]. Therefore, there is an urgent need for renewable energy to alleviate the present energy and environmental problems. Solar energy has received much attention due to its environmentally friendly and inexhaustible characteristics [3,4].

Solar energy can be converted into chemical energy and stored in fuel molecules via photocatalytic reactions [5]. Similarly, organic pollutants can also be removed or transformed into value-added chemicals by photocatalysis [6,7]. Thus, photocatalysis has become a promising strategy to resolve the present energy and environmental problems, considering its mild reaction conditions, low cost, environmental friendliness, and other advantages. Photocatalysts play an essential role in photocatalytic reactions. Generally, the photocatalysts should be sensitive to a wide spectrum of light and have excellent light-harvesting ability [8], efficient photoelectric separation and transfer [9], appropriate electron energy levels [10], and high photostability. To meet these requirements, metal oxides [11,12], organic dyes [13,14], covalent organic frameworks [15,16,17], metal-organic frameworks [18,19,20], carbon nitride materials [21], quantum dots [22], and other photocatalysts have been used in photocatalytic reactions. Among them, organic photocatalysts attracted remarkable attention owing to their wide light absorption spectrum and tenability in structure and functionality [23]. Porphyrin and its derivatives possessing a conjugated macrocycle structure similar to chlorophyll in natural photosystem and a high molar absorption coefficient were widely investigated in photocatalysis.

Porphyrins are heterocyclic molecules composed of a core porphin ring and different peripheral substituents [24]. Various porphyrin derivatives can be obtained by changing the outer groups or centrally coordinated metal ions [25,26]. As dye molecules, porphyrins have the following characteristics: (1) Most of them have high molar absorbance coefficients and excellent absorption spectra in the Soret-band and Q-band [27]. (2) The large π-conjugation system and unique planar structure endow them with intermolecular π-π stacking tendency and charge transport probability [28]. (3) Facile modification of the peripheral groups of porphyrins and the diversity of central metal species confer them with tunable band structures and optoelectronic properties [29]. These ensure porphyrins have light sensitivity and self-assembling capacity and facilitate their application in photocatalysis [30,31,32], photodynamic therapy [33,34], sensing, and other fields [35].

However, in spite of such advantages, the inherent gap between the Soret-band and Q-band of porphyrin monomers is exactly located in the strongest output range of the sun’s irradiance spectrum, resulting in relatively lower solar energy utilization efficiency [36]. More importantly, free porphyrin molecules or disordered accumulation of porphyrin tend to limit exciton coherence to a smaller region, resulting in ineffective electron excitation [37]. Additionally, expensive and complex noble-metals including Pt nanoparticles and Ru complexes, are usually required to facilitate charge separation and overcome the unmatched energy levels [38,39]. This undoubtedly increases the cost of the photocatalytic system and the difficulty of subsequent purification. In addition, porphyrins, as organic photocatalysts, are easily photobleached, which limits their continuous catalytic ability and increases the difficulty of recycling.

One approach to improve the light-harvesting performance of porphyrin monomers is to introduce other chromophores in porphyrins via chemical synthesis [40,41]. Although the light-response performance is improved, some new problems, including a complex preparation process, insufficient stability of synthetic materials, and a cumbersome purification process, are also involved [42]. Another alternative method is to form supramolecular assemblies, which can also improve the utilization of solar energy, and at the same time, the charge recombination can be effectively inhibited. Thus, fast charge separation and transfer can be obtained by improving electron delocalization in a larger assembling zone [43]. Moreover, the supramolecular assembling strategies are simple and easy to operate in a liquid phase [44]. Herein, we reviewed the design, preparation, stability, and relationships between the structure and property of porphyrin supramolecules and introduced their application in hydrogen evolution, carbon dioxide fixation, coenzyme NADH generation, and environmental regulation. We focused on the intrinsic mechanism of charge separation and transfer within various supramolecules and attempted to build relationships among the arrangement of porphyrin, charge transfer pathway, and final photocatalytic performance. We wish to increase the understanding of authors in porphyrin-based supramolecules and broaden their application in photocatalysis.

## 2. Porphyrin Supramolecules

**Figure 1:**
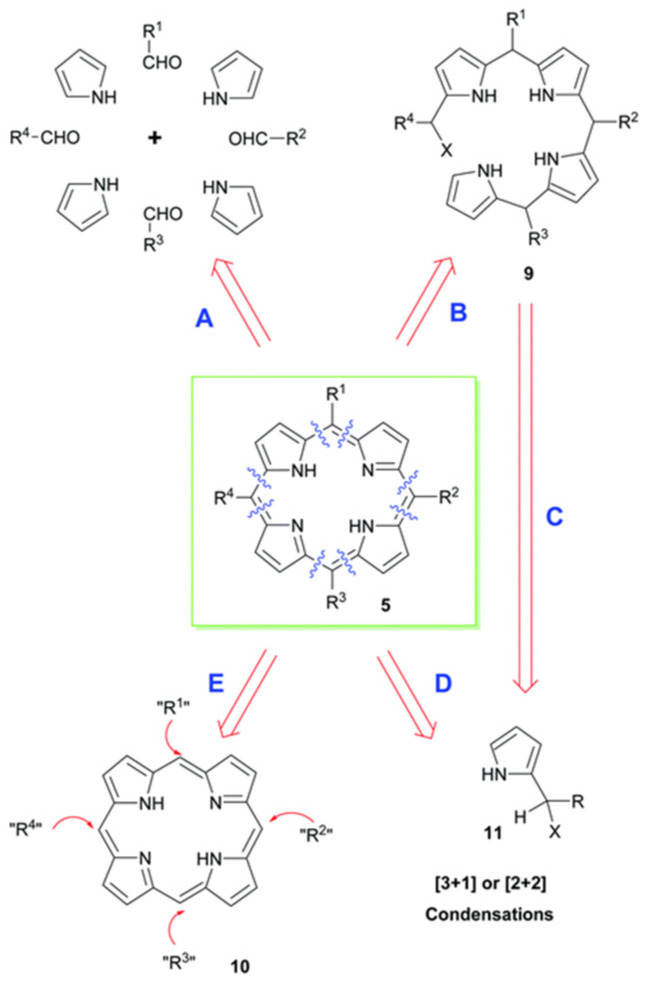
Some approaches used to build up porphyrins [24].

Porphyrin supramolecules come from the ordered organization of porphyrin monomers. There are different methods to synthesize porphyrins [24]. As summarized in Figure 1, pathway A decomposed the parental structure into four pyrrole units and coupled them with appropriate aldehydes under acid catalysis, followed by oxidation of non-aromatic porphyrinogen intermediates to target porphyrins; Pathway B depends on the total synthesis of open-chain alkane 9, followed by a ring closure reaction; Pathway D uses various 2-pyrrole component precursors 11 to achieve similar polycondensation; Pathway E utilizes functional group interconversion and novel coupling reactions to introduce the necessary side chains.

Due to their π-π conjugated structure and abundant peripheral groups, porphyrin molecules tend to self-assemble in aqueous solution through π-π stacking, electrostatic interaction, hydrophobic interaction, hydrogen bonding, coordination, metal complexation, and other non-covalent interactions [45]. Similar to chloroplasts in natural photosystems, porphyrin assemblies can also complete the functions of light capturing, light-to-electric conversion, and light irradiation to chemical energy transformation in artificial photosystems [46]. Importantly, porphyrin supramolecules usually exhibit better performance in light harvesting and photocatalytic performance.

### 2.1. Supramolecular Structure

**Figure 2:**
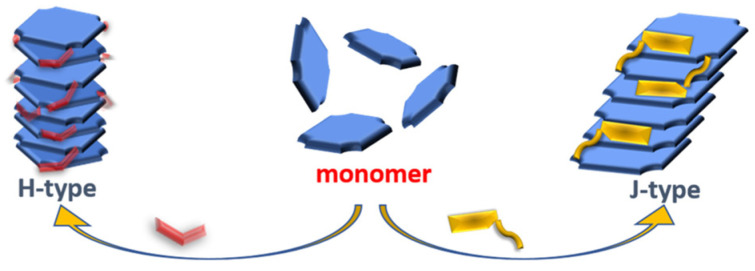
Diagram of J-type and H-type porphyrin aggregates [47].

Generally, porphyrin molecules form two typical arrangements during the aggregation process, namely J-type and H-type aggregates (Figure 2) [47]. The two aggregation modes show significant differences in molecular arrangement and spectral characteristics. In H-type aggregates, porphyrin molecules are tightly packed *face-to-face*. This arrangement leads to the enhancement of π-π interaction between molecules and the increase in electron cloud overlap, which leads to energy level splitting and the change of energy band structure. As reflected in the absorption spectrum, H-type aggregates exhibited an obvious blue shift, that is, the absorption peak shifted to the short wavelength direction compared with the free monomers. This blue shift phenomenon is usually accompanied by an increase in absorption intensity and a narrow spectral bandwidth, indicating that H-type aggregates have unique advantages in light absorption characteristics.

In contrast, the porphyrin molecules of J-type aggregates are arranged *side-by-side*, similar to that of chlorophyll molecules in natural photosynthetic systems. In J-type aggregates, the π-π interaction between porphyrin molecules is relatively weak, resulting in a significant redshift in the absorption spectrum, that is, the absorption peak shifts toward the long wavelength. The redshift phenomenon enhances the absorption capacity of J-type aggregates in the visible light range, especially its maximum absorption, which appears in the strongest range of the full spectrum irradiation (about 500 nm), which greatly improves the utilization of visible light [45], which is of great significance for the application of photocatalysis and optoelectronic devices.

Due to the different arrangement of porphyrin molecules in J-type or H-type aggregates, their band structure and exciton dynamics show significant differences [48]. Specifically, the band structure of H-type aggregates is relatively tight, and the exciton localization is strong, while the band structure of J-type aggregates is relatively loose, and the exciton migration distance is longer. Studies have shown that the ordered arrangement of porphyrin molecules in J-type aggregates contributes to the efficient migration of excitons within the aggregates, resulting in efficient electron transport efficiencies [49]. This efficient electron transport characteristic enables J-type aggregates to perform better in photocatalysis and photoelectric conversion processes. Therefore, J-type aggregates are considered to be a better choice, with broad application and research value.

### 2.2. Construction of Porphyrin Supramolecules

The construction of porphyrin supramolecules has attracted great interest in photocatalysis owing to their fascinating properties. As is known, peptides and proteins act as scaffolds to regulate chlorophyll molecules to form an ordered array in natural photosynthetic systems and exhibit efficient light harvesting efficiency. Inspired by the natural photosystem, various organic templates were used to regulate the structure of the aggregates and the self-assembling behavior of porphyrin molecules, such as polymers, peptides, organic molecules, metal ions, etc. [50,51,52], resulting in porphyrin-based nanomaterials with diverse morphologies, sizes, and excellent optoelectronic properties.

#### 2.2.1. Self-Assembly of Porphyrin Molecules

Porphyrin molecules can spontaneously form highly ordered functional nanostructures through non-covalent interactions due to their unique conjugated structure and abundant optoelectronic properties. For example, porphyrin monomers can self-assemble into nanostructures by reprecipitation, ion self-assembly, coordination polymerization, etc. [53]. The synergistic effect among the orderly arranged molecules can significantly improve their light absorption, charge transport, and catalytic performance [54,55]. It is possible to regulate the morphology, size, and spatial arrangement of the assemblies by precisely selecting the functional groups and introducing specific auxiliary components.

**Figure 3:**
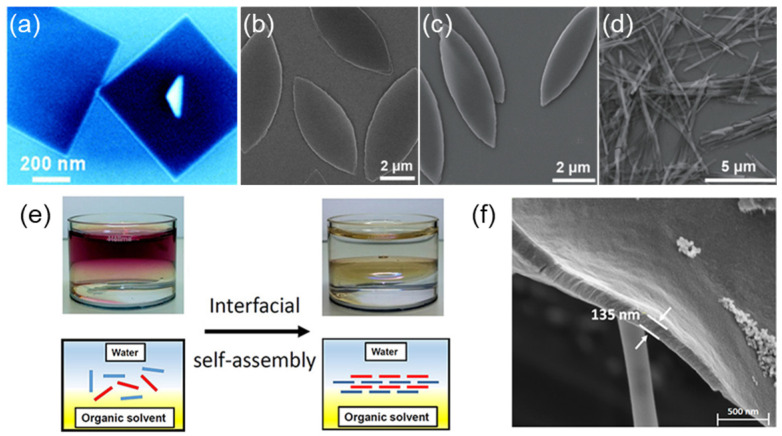
(**a**) TEM image of SnPyTriPP nanosheets on a Si(100) substrate [56]; (**b**) TEM image of PdTCPP nanoleaves [57]; (**c**) TEM image of PtTCPP nanoleaves; (**d**) SEM image of PdTCPP nanoribbon [58]; (**e**) ZnTCPP self-assembles to highly ordered nanofilms; (**f**) SEM image of a film of ZnPor-INs transferred from the water [59].

Porphyrin molecules can self-assemble into various layered self-assembled structures, such as nanosheets, nanorods, nanoleaves, nanofiber bundles, and other morphologies. Wang et al. [56] successfully prepared porphyrin nanosheets by self-assembling SnIV5-(4-pyridyl)-10,15,20-triphenyl porphyrin (SnPyTriPP) in water using the reprecipitation self-assembly method (Figure 3a). Cao et al. [57] prepared well-defined spindle-like nanoleaves from self-assembled Pd (II) tetra-(4-carboxyphenyl) porphyrin (PdTCPP) by a mixed solvent method (Figure 3b). Bai et al. [58] prepared regular nanocrystals with different ordered superimposed structures through non-covalent bond interactions of tetrad (4-carboxyphenyl) porphyrin (PtTCPP) (Figure 3c,d). These ordered self-assembled nanostructures have potential applications in electronics, photonics, and catalytic systems. Interestingly, researchers [59] constructed highly ordered nanofilms at the interface of water and organic solvents through hydrogen bonding and hydrophobic interactions of (ZnTCPP) (Figure 3e,f). This method was conducted at mild conditions (pH 5.8), which prevents the expulsion of metal ions from the metalporphyrin at acidic conditions. Moreover, the self-assembly process does not depend on additives such as surfactants and external stimuli. This provides an alternative approach to spontaneous light-harvesting antennas.

#### 2.2.2. Co-Assembly of Porphyrin Molecules

Although the self-assembly of porphyrin molecules improves the photocatalytic performance to a certain extent, the stability of porphyrin self-assemblies is easily affected by the external environment (such as temperature, humidity, solvent, etc.), resulting in limited stability and poor reusability [60,61]. Additionally, porphyrin self-assemblies are easily photodegraded under long-term light irradiation due to their failed delivery of the photoinduced “hot electrons”, resulting in the decline of photochemical and photophysical properties and further limiting their application in photocatalysis [62,63]. Therefore, the co-assembly strategy was attempted to construct stable and efficient porphyrin-based photocatalysts.

Based on their unique conjugated structure, porphyrin can co-assemble with porphyrin or other molecules through various non-covalent interactions to form supramolecular systems with highly ordered structures and specific functions [64,65,66]. Co-assembled materials usually exhibit superior properties than single materials. Cheap and abundant raw materials can also be used to prepare high-performance composite materials to reduce production costs [67]. For example, in organic solar cells, the efficiency of light absorption and charge separation can be improved through the co-assembling of porphyrins, thereby improving the photoelectric conversion efficiency [68,69]. In the photocatalyst design, mesoporous materials with high specific surface area and abundant active sites, as well as catalytic activity, can be prepared through the co-assembly method [70].

**Figure 4:**
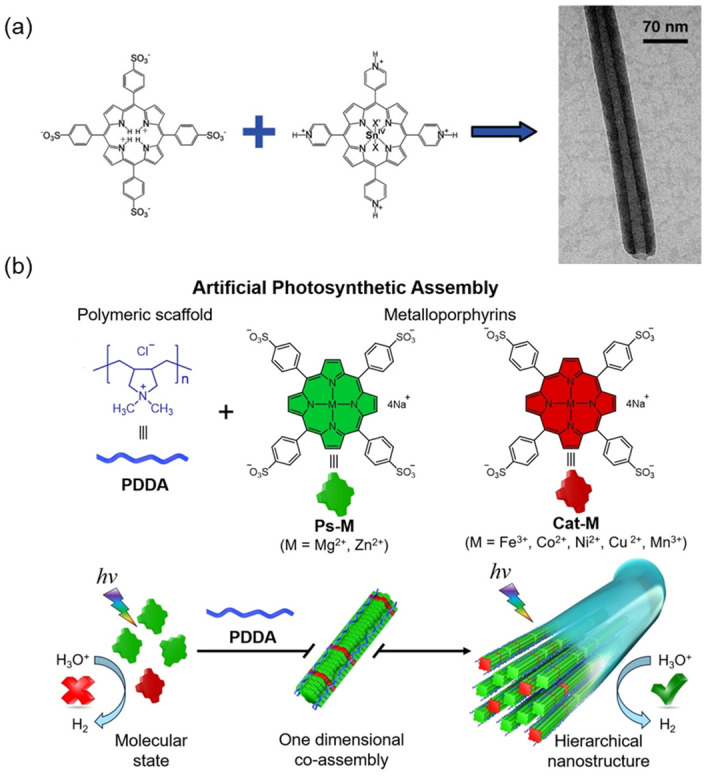
(**a**) Co-assembly of oppositely charged porphyrins to form porphyrin supramolecular nanotubes [71]; (**b**) Schematic diagram of metal porphyrins and PDDA co-assembled into multi-level supramolecular nanostructures [72].

Wang et al. [71] prepared porphyrin nanotubes through electrostatic interactions of oppositely charged porphyrin monomers (Figure 4a). The prepared nanotubes have a diameter of 50~70 nm, a length of several microns, and a wall thickness of ~20 nm. The porphyrins in the nanotubes are stacked in a J-aggregating manner, showing typical absorption peaks at 496 and 714 nm in the UV-Vis spectra, significantly redshifted compared to that of the porphyrin monomers. Mg and Zn porphyrins are widely recognized as effective photosensitizers with excellent photon capture capabilities, while other transition metals, such as Fe, Co, Ni, Cu, and Mn coordinated porphyrins, are usually believed to be photocatalysts. Co-assembly of a photosensitizer with excellent photon-capturing ability and a photocatalyst with high catalytic performance can efficiently transfer light energy from the photosensitizer to the catalytic center. Inspired by the assembly of bile proteins in phycobilins, supramolecular nanostructures were constructed by co-assembling the porphyrin photosensitizer tetrad—(4-sulfonyl phenyl) magnesium porphyrin (MgTPPS) and porphyrin catalyst (CoTPPS) on artificial polyelectrolyte (PDDA) scaffolds (Figure 4b) [72]. The supramolecular complex exhibited excellent photostability and high photocatalytic activity, in which the hydrogen production rate reached 793.2 μmol g^−1^ h^−1^, which is 23 times higher than that of free molecular controls.

#### 2.2.3. Surfactant-Assisted Assembly of Porphyrin Molecules

**Figure 5:**
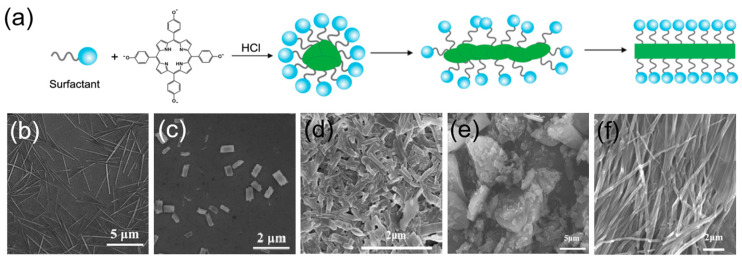
(**a**) Nucleation and growth of porphyrin nanostructures; (**b**) SEM image of CTAB-THPP nanowires; (**c**) SEM image of CTAB-THPP nanorods [74]; (**d**) SEM image of CTAB-TCPP aggregates; (**e**) SEM image of TCPP powder; (**f**) SEM image of TCPP aggregates [75].

Surfactant-assisted assembly of porphyrins has attracted extensive attention due to their excellent reproducibility, simple operation, hierarchical assembling structure, and good adjustability [58,73]. The arrangements of porphyrin molecules in the surfactant-assisted nanostructures can be well controlled and endowed with tunable photocatalytic activity. For example, protonated porphyrin molecules (H_2_THPP^2+^) were encapsulated in the hydrophobic cavity of the acidic surfactant micelles (Figure 5a) and self-assembled into various nanostructures by adjusting the pH value and corresponding internal spatial arrangements (Figure 5b,c). The nanostructures exhibited a high hydrogen evolution rate (19.5 mmol g^−1^ h^−1^), while the photocatalytic activity of the porphyrin monomer was negligible [74].

TCPP was self-assembled into regular nanostructures in the presence of cetyltrimethylammonium bromide via an acid-base neutralization reaction (Figure 5d) [75]. Compared with TCPP monomers (Figure 5e,f), the Soret-band of the supramolecules redshifted to 435 nm, and the Q band also shifted to the longer wavelength, suggesting that the TCPP molecules arranged in a J-type pattern. The supramolecules showed a 94% degradation of phenol, while no significant degradation signal was observed for the unassembled TCPP powder under similar reaction conditions.

#### 2.2.4. Peptide-Induced Assembly of Porphyrin Molecules

**Figure 6:**
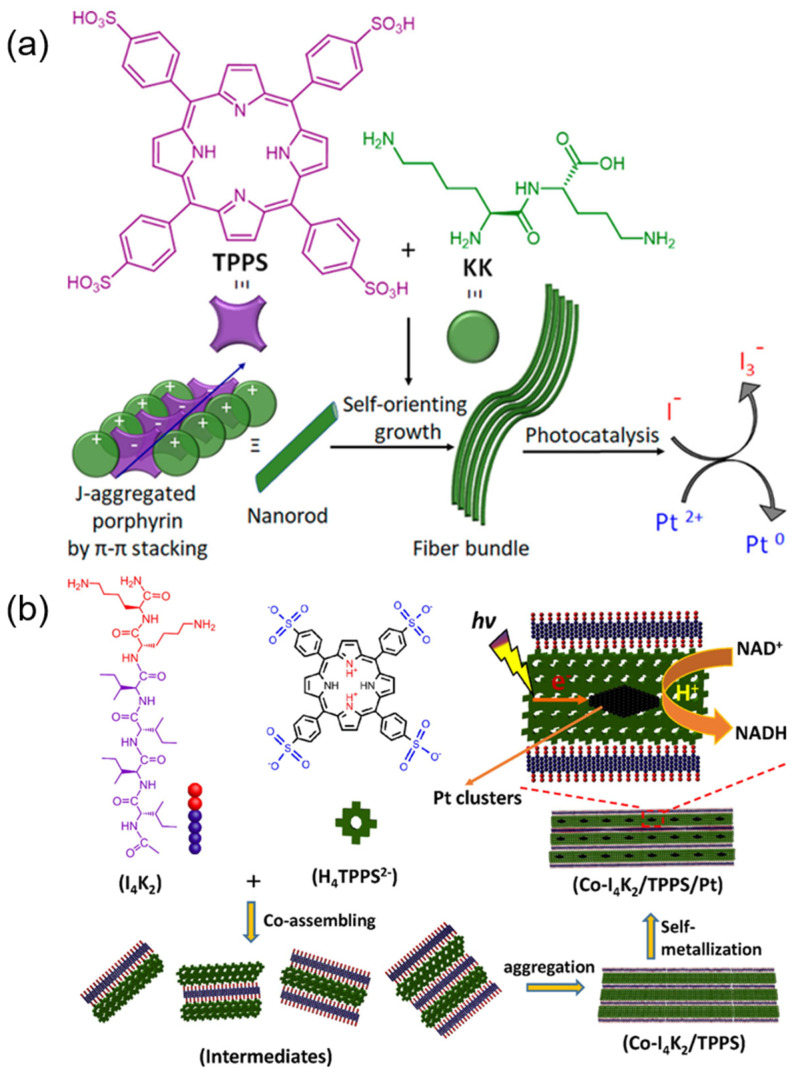
(**a**) Schematic diagram of the co-assembly of TPPS catalyst and dipeptide (KK) into fiber bundles and photocatalysis [76]; (**b**) Schematic design and construction of light capture antenna (Co-I_4_K_2_/TPPS/Pt complex) [36].

The porphyrin-polypeptide (protein) assembling systems are ubiquitous, and it is an effective way to construct an artificial photosynthetic system by using its special molecular recognition and self-assembling ability. Liu et al. [76] took (TPPS) and dipeptide (KK) as assembly units to form long-range arranged porphyrin J aggregates (Figure 6a). The porphyrin-peptide assembly exhibited excellent photostability, enhanced charge separation properties, and improved photocatalytic oxidation performance. The introduction of KK allows TPPS to self-assemble into J-aggregates at mild conditions and endows them with good optoelectronic properties and stability.

Wang et al. [77] prepared a hierarchical I_4_K_2_/TPPS/Pt complex by integrating light collection and electron charge separation units through a three-step method of molecular recognition, layer assembly, and self-metallization. The complex showed extraordinary sensitivity for weak light, in which visible light could be transformed to chemical energy and stored in NADH molecules. This is mainly attributed to their hierarchical assembly structure and special coupling of light harvesting and electron transfer units. Subsequently, Wang et al. [43] systematically explored the factors that induce porphyrin (TPPS) to form J-aggregates on self-assembling peptide (I_4_K_2_). With the increase in acidity of the solution, the degree of protonation of core nitrogen atoms and the cationic peptide self-assemblies partially neutralize the negative charges of TPPS, which helps to reduce the intermolecular electrostatic repulsion of TPPS, resulting in the formation of ordered porphyrin J-aggregates on the peptide templates. Due to the introduction of the positive peptide templates, regular J-aggregates formed under mild conditions, which greatly optimizes the preparation condition of porphyrin J-aggregates and expands its application fields. Finally, Wang et al. [36] prepared a layered photoactive complex (Co-I_4_K_2_/TPPS/Pt) (Figure 6b) through a co-assembly strategy, showing excellent photocatalytic properties and remarkable sustainability under strong light irradiation (35,000 lx) and extraordinary sensitivity to low light (700 lx), a promising step for artificial light systems.

#### 2.2.5. Polymer-Induced Assembly of Porphyrin Molecules

**Figure 7:**
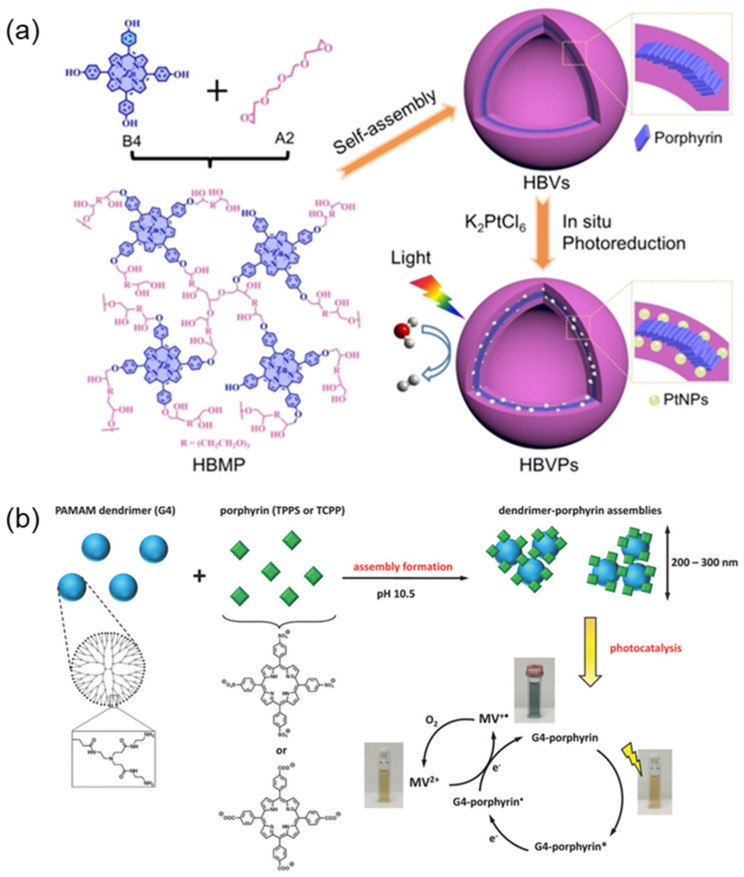
(**a**) Schematic diagram of a supramolecular membrane photocatalytic system based on HBVP hybrid vesicles [80]; (**b**) Dendritic macromolecule-porphyrin self-assembly and photocatalytic reduction in methyl violet (MV) [81].

In a polymer-induced artificial supramolecular photosystem that mimics a natural photosynthetic system, the polymer functioned as that of thylakoid membrane components in plants. The polymer-induced porphyrin self-assemblies have some advantages in the design of photocatalytic systems. They have hierarchical chemical and spatial structures, in which the interaction of porphyrin and polymer affected the formation of porphyrin assemblies [78,79]. A porphyrin-based bionic vesicle photocatalyst was constructed by combining the synthesis of hyperbranched multi-porphyrin polymer (HBMP) vesicle membranes with porphyrin molecules (THPP) and platinum nanoparticles (PtNPs) (Figure 7a) [80]. The photocatalyst exhibited high photocatalytic activity and excellent stability due to the orderly arrangement of porphyrin units, which are helpful in promoting the photogenerated electron-hole separation. Anne Kutz et al. [81] designed a functional self-assembled nanostructure based on porphyrin molecules and the polyamide (PAMAM) dendritic polymer (Figure 7b), which showed high photoelectric efficiency compared to unbound porphyrins, showing a six-fold increase in the photocatalytic reduction rate of methyl viologen (MV).

#### 2.2.6. Metal Ion-Induced Assembly of Porphyrin Molecules

**Figure 8:**
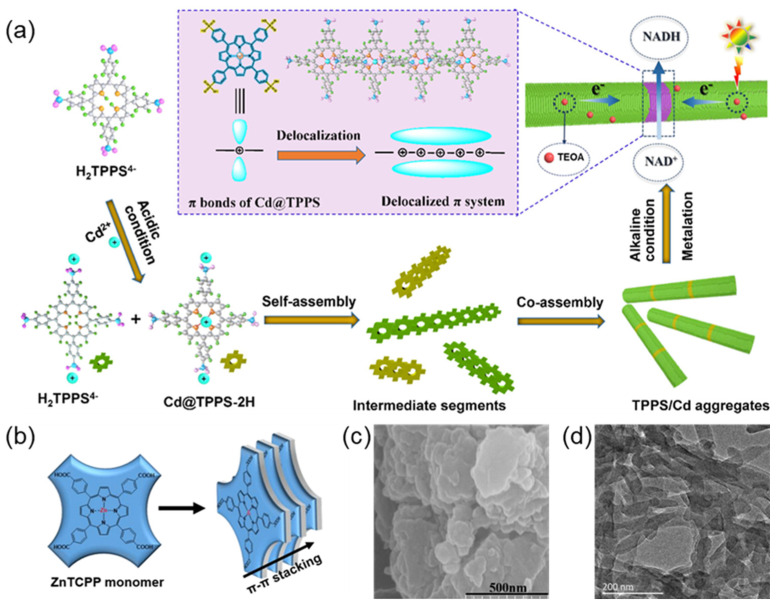
(**a**) Schematic diagram of the design and construction of a light-harvesting antenna based on the absence of precious metal porphyrins [82]; (**b**) ZnTCPP self-assembly diagram via π-π interactions; (**c**) SA-ZnTCPP SEM diagram; (**d**) SA-ZnTCPP TEM diagram [83].

Over-reliance on expensive electronic media (e.g., rhodium complexes) and electron separators (e.g., Pt nanoparticles) increases the cost and purification difficulty in the existing artificial photosynthetic systems. The coordination of non-precious metal ions in porphyrin rings is expected to alleviate such problems. Wang et al. [82] designed a noble metal-free porphyrin-based light-capturing antenna (Figure 8a) with a *side-by-side* arranging pattern induced by Cd^2+^ ions. The Cd^2+^ ions coordinated antenna showed significant activity and excellent stability owing to the promoted charge separation and transfer. This work represents an essential step in constructing precious metal-free porphyrin-based artificial light systems. Metalloporphyrin complexes, in nature, are assembled with proteins or biofilms before they perform their functions. Thus, the investigation of the supramolecular self-assembly of metalloporphyrins become a hot topic in biomimetic chemistry. Zhu et al. [83] prepared a self-assembled nanosheet structure through π-π interactions of tetrad (4-carboxyphenyl) zinc-porphyrin supramolecule (SA-ZnTCPP) (Figure 8b–d). Compared with the metal-free porphyrin assemblies, the photocatalytic activity of the SA-ZnTCPP in hydrogen production is about 85 times higher due to their higher conduction band caused by the insertion of zinc ions in porphyrins.

The above results show that the self-assembly of porphyrins can be achieved and regulated by changing peripheral groups [84], the introduction of structural regulators [85], metal complexation [86], and changing the solution [87] to provide anchor sites and/or weaken the intermolecular repulsion. The comprehensive interaction of light traps, biomolecules, and other functional units drives the formation of artificial photosynthetic systems. However, organic molecules that are insensitive to light and do not conduct electricity will inhibit the transfer of the photoexcited charges, lowering the photocatalytic efficiency and limiting their further applications. With the photocatalytic reaction step forward, we can improve photocatalytic activity in two ways: to improve the light-absorbing capacity of the photocatalyst and to accelerate the separation and transfer of photogenerated charges [88]. The increase in photocatalytic capacity usually requires a decrease in the bandgap, which will decrease their reducing capacity (reduced conduction band) or oxidizing capacity (increased valence band). As is known, approaches to improve the charge separation and transport efficiency play a vital role in boosting both the photocatalytic oxidation and the photocatalytic reduction efficiency [89].

## 3. Charge Separation and Transport Regulation

### 3.1. Construction of Heterostructures

Porphyrins have shown essential applications as photocatalysts in energy conversion and environmental governance, but the rapid recombination of electron-hole pairs limited their performance. The photo-excited electrons and holes are easily recombined, resulting in low photocatalytic efficiency [90]. As is widely accepted, a narrow bandgap in a photocatalyst helps to expand its light absorption spectrum. However, paradoxically, it would decrease the oxidation or reduction capacity by narrowing the bandgap because the redox performance is closely related to its conduction band (CB) or valence band (VB) position [91]. The introduction of heterojunctions provides a promising way to solve this problem. The heterojunction is an interface structure composed of two different semiconductor materials that can effectively promote the separation of electron-hole pairs through energy level matching and interface effects [92].

**Figure 9:**
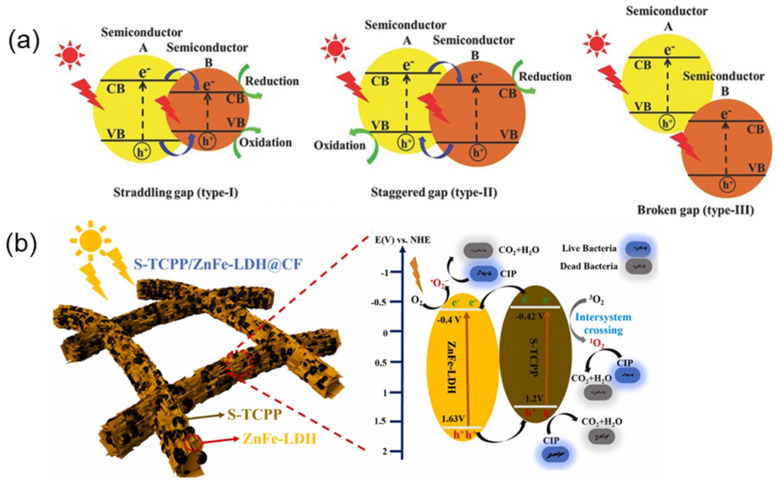
(**a**) Schematic diagram of three different types of heterojunction electron-hole pair separation [93]; (**b**) Photocatalytic charge transfer mechanism of S-TCPP/ZnFe-LDH heterojunction [94].

Currently, several heterojunctions have been developed, as shown in (Figure 9a) [93]. Type I heterojunctions are unsuitable for facilitating electron-hole pair separation because their band structure is not interlaced. The band structure of type III heterojunctions does not overlap, in which charge transfer across the interface is difficult. In contrast, type II heterojunctions have attracted great concern due to their staggered band structures. The photogenerated electrons easily transferred from A to B, while the photogenerated holes transferred in the opposite direction, which effectively promotes the separation of electron-hole pairs and improves the photocatalytic efficiency. Xue et al. [94] synthesized supramolecular porphyrin/ZnFe layered double hydroxides (S-TCPP/ZnFe-LDH) hybrids with type II heterojunctions through an in-situ self-assembling method (Figure 9b). Charge carriers can efficiently migrate across the heterogeneous interfaces due to their staggered band structure. The photocatalyst with Type II heterojunction exhibits a higher photocatalytic degradation efficiency compared to the pristine photocatalyst (ZnFe-LDH).

**Figure 10:**
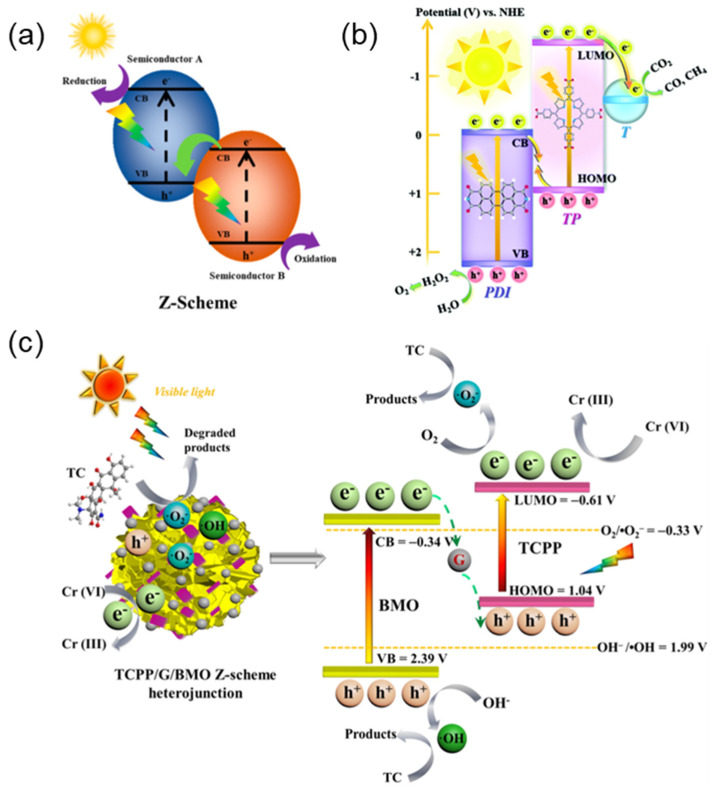
(**a**) Z-type heterojunction electrons-Schematic diagram of hole pair separation [97]; (**b**) Photocatalytic charge transfer mechanism of T-TP/PDI heterojunction [98]; (**c**) Photocatalytic mechanism of TC degradation on TCP/G/BMO under visible light irradiation [99].

However, for the photocatalysts with Type II heterojunctions, the reduction reaction occurs at the semiconductor with lower CB while the oxidation reaction occurs at the semiconductor with upper VB, which will decrease the oxidation or reduction ability, although the charge recombination is suppressed [95]. Therefore, balancing the charge separation efficiency and redox ability is crucial for the design of such photocatalysts. Bard [96] proposed a Z-type heterojunction model. Unlike type II heterojunctions, the photoinduced electrons in Z-type heterojunctions tend to migrate from the CB of semiconductor B to the VB of semiconductor A (Figure 10a) [97], which can improve the charge separation efficiency on the basis of retaining the original redox ability. Wang et al. [98] prepared Z-type heterojunctions (T-TP/PDI) of TiO_2_ and tetrad (4-carboxyphenyl) porphyrin/perylene diimide (Figure 10b). Both PDI and TP generated electron-hole pairs under visible light irradiation. The photogenerated electrons on the CB of PDI transferred to the HOMO of the TP, and the electrons separated by TP space further transferred to TiO_2_. The photocatalytic activity improved about 10 times compared to the original PDI. Liu et al. [99] designed a new Z-type heterojunction (TCPP/G/BMO) considering the matched band structure of bismuth molybdate (Bi_2_MoO_6_) and porphyrin (TCPP) and the excellent conductivity and sunlight collection ability of graphene quantum dots (GQDs) (Figure 10c). The electron-hole separation efficiency was significantly improved, and at the same time, the high redox capacity was retained, resulting in excellent photocatalytic performance in tetracycline (TC) removal.

**Figure 11:**
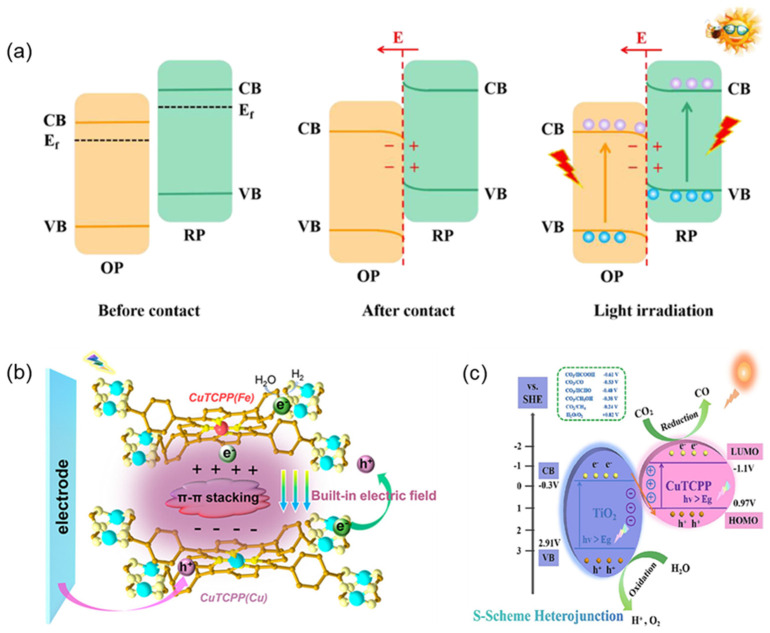
(**a**) The formation process of S-type heterojunction and the migration path of photogenerated carriers [102]; (**b**) Schematic diagram of the interface structure of bimetallic porphyrin heterojunctions [104]; (**c**) Photocatalytic charge transfer mechanism of CuTCPP/TS heterojunction [105].

The S-type heterostructure also enables the spatial separation of photoinduced electron-hole pairs and maintains sufficient redox capacity [100,101]. Compared to Z-type heterojunctions, Fermi level or bending information in S-type heterojunctions was taken into consideration. The Fermi level and CB position of the reduction photocatalyst (RP) in the S-type heterojunction is higher than that of the oxidation photocatalyst (OP), so the photogenerated electrons in the CB of the RP transfer to the CB of the OP when the two semiconductors contact. The RP loses electrons, causing the band to bend upward at the interface. Conversely, the bands at the OP interface bent downward. At the same time, a built-in electric field formed at the interface to guide electron transfer from the RP to the OP. The photogenerated electrons in the CB of OP recombine with the holes in the VB of RP through the internal electric field. The residual photogenerated holes in the VB of OP and the photogenerated electrons in the CB of RP undergo redox reactions in the subsequent time (Figure 11a) [102].

The S-type heterojunctions enable efficient separation and migration of photoinduced electrons and holes and endow them with strong photocatalytic redox ability [103]. Liu et al. [104] prepared bimetallic porphyrin J-aggregates by electrostatic self-assembly (Figure 11b). The ultra-thin nanosheet structure shortened the carrier transport distance, the similar porphyrin structure provided suitable interface matching through π-π conjugations, and the S-type heterojunctions adjusted by the porphyrin central metal generated an intensive built-in electric field. Carrier separation and transfer can be efficiently improved. Zhang et al. [105] constructed an S-type heterojunction (CuTCPP/TS) between TiO_2_ nanosheets and CuTCPP (Figure 11c), which broadened the light response range, improved the charge separation efficiency and redox potential, and thus significantly enhanced their photocatalytic activity.

### 3.2. Construction of Donor-Acceptor (D-A) Structure

The design and construction of the internal structure of supramolecular materials is an effective strategy to optimize their optoelectronic properties. Donor-acceptor (D-A) systems have attracted attention due to their potential application in the preparation of optoelectronic materials and devices [106]. Interactions between electron-rich and electron-deficient moieties allow the formation of long-range ordered electron transfer channels, which inhibits the photoinduced charge recombination [107]. Porphyrins are an ideal donor moiety in the donor-acceptor electron system (D-A) due to their strong electron-donor ability and extended π-conjugated system. They can also function as acceptor moieties via the coordination of metal ions with the porphyrin core to provide a Lewis acid site [108]. Additionally, a broadened spectral response range can also be obtained when a porphyrin-based donor-acceptor structure is introduced [109].

**Figure 12:**
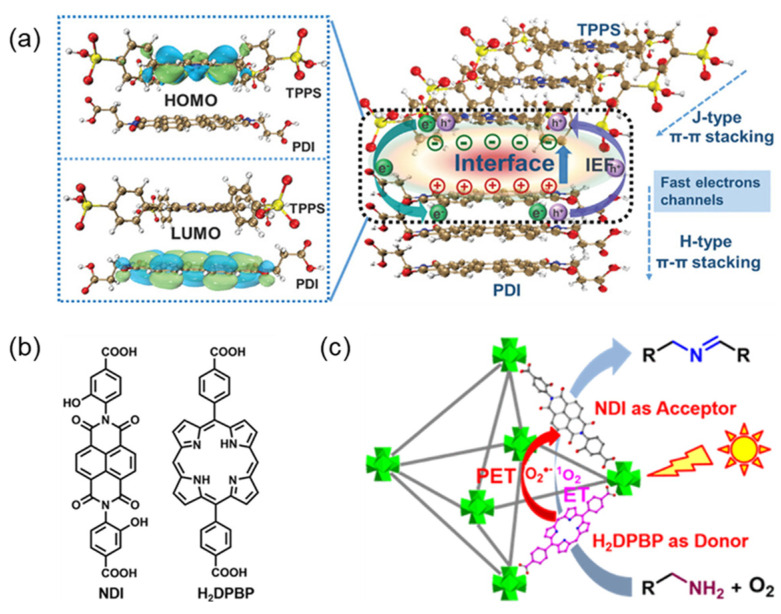
(**a**) Schematic diagram of the photocatalytic mechanism of co-assembly supramolecular TPPS/PDI [109]; (**b**) Chemical structures of ligands NDI and H_2_DPBP; (**c**) Schematic diagram of the mechanism of Zr-NDI-H_2_DPBP-MOF photocatalytic amine coupling reaction [111].

Yang et al. [109] successfully constructed TPPS/PDI complexes with D-A structures (Figure 12a), in which their LUMO and HOMO at the interface are located on PDI and TPPS, respectively, indicating that TPPS has excellent electron donor characteristics and PDI has electron acceptance characteristics. The photocatalytic efficiency in hydrogen evolution is about 9 times that of pure TPPS. The short-range J-type aggregation of TPPS well simulated the chlorophyll in nature, while the long-range H-type aggregation of PDI can provide long-range electron delocalization, and the integration of the two types of π-π stacking can effectively promote the charge separation and transfer. Souza et al. [110] constructed a complex including a photosynthetic antenna and a reaction center, in which boron bromide (BDP) absorbed light energy, excited electrons transferred from zinc porphyrin (ZnP) donor to fullerene electron acceptor, and electron excitation energy was converted into chemical energy simultaneously.

Porphyrin-based metal-organic frameworks (MOFs) based on donor-acceptor systems have also shown excellent performance in photocatalysis. Xu et al. [111] constructed a MOF (Zr-NDI-H2DPBP) photocatalyst using 5,15-bis (p-benzoate) porphyrin (H_2_DPBP) as an electron donor and naphthalene diimide (NDI) as an electron acceptor (Figure 12b,c). The MOF photocatalyst showed high charge separation efficiency and excellent photocatalytic performance. Similar donor-acceptor structures are critical in designing metal-organic and covalent organic frameworks for photocatalysts [112,113].

### 3.3. Introduction of Co-Catalysts

The performance of photocatalysts is far from satisfactory, although great progress has been achieved during the past few years. On one hand, electron-hole pairs are easy to recombine before migrating to the surface for reaction. On the other hand, slow surface reactions dissipate charge efficiently [114]. To better “transport” electrons to the surface, metals, especially precious metals such as Pt, are used as co-catalysts. Precious metals function as electron separators to rapidly separate electrons from the photogenerated electron-hole pairs and provide efficient proton reduction sites to facilitate reduction reactions. As a co-catalyst, Pt can also adjust the band structure of the photocatalyst, change the light absorption and photoelectron transport properties, solve the problem of charge recombination, and accelerate the charge transfer [80].

Layered porphyrin-based light-capturing antennas (Fmoc-ChaChaGK/TPPS/Pt and Fmoc-FFGK/TPPS/Pt) were constructed by a three-step method of peptide self-assembly, surface porphyrin aggregation, and self-metallization [115]. Compared with the sample before metallization, the metalized complex exhibited higher photocurrent responses under light irradiation. The role of Pt nanoparticles in separating and transferring charges was demonstrated. The results provided new insights into the design and construction of light-responsive materials in integrated light-harvesting antennas and artificial light systems. Liu et al. [116] constructed light responsive fiber bundles via short peptide-induced porphyrin self-assembly and subsequently in-situ mineralized with TiO_2_ and Pt nanoparticles on the surface of the fiber bundles. This photocatalytic system showed high visible light capturing, electron transport, and energy transfer efficiency.

## 4. Stability

The stability of a photocatalyst is a critical factor in determining its usefulness and long-term effect. The stability not only affects its durability in the environment but also determines the breadth and depth of its application in industry and scientific research [117,118]. Porphyrin molecules often suffer from photobleaching, which reduces their light-capturing ability. This is mainly caused by the “hot electrons” that cannot transfer in a timely manner. The chemical and physical stability of porphyrin-based photocatalysts can also be enhanced by improving the charge transport efficiency in addition to the photoelectric conversion efficiency [60,119]. For example, an artificial supramolecular system formed by anionic metalloporphyrin co-assembled with cationic metalloporphyrin on polyelectrolyte scaffolds showed significantly improved resistance to light irradiation compared to the porphyrin molecules [120]. The enhancement in stability was attributed to the efficient energy and charge transfer between the photosensitizer and photocatalyst. Efficient electron transport can reduce energy consumption and possible decomposition due to electron-hole recombination, enhancing the material’s stability over long-term use. Therefore, strategies that help to improve the charge separation efficiency are conducive to the stability of the photocatalysts.

Additionally, the porphyrin supramolecules often exhibited poor photostability when they were dissolved by the solvent [121,122]. Therefore, improving the stability of porphyrin supramolecules, especially under extreme conditions such as high temperature, intense sunlight, and chemical corrosion, has become an essential topic in laboratory and industrial applications. There are many research studies to improve the stability of porphyrin supramolecules by chemical modification, core metallization, and adjustment of internal spatial structure in the self-assembling process [117,123,124,125].

### 4.1. Chemical Modification

**Figure 13:**
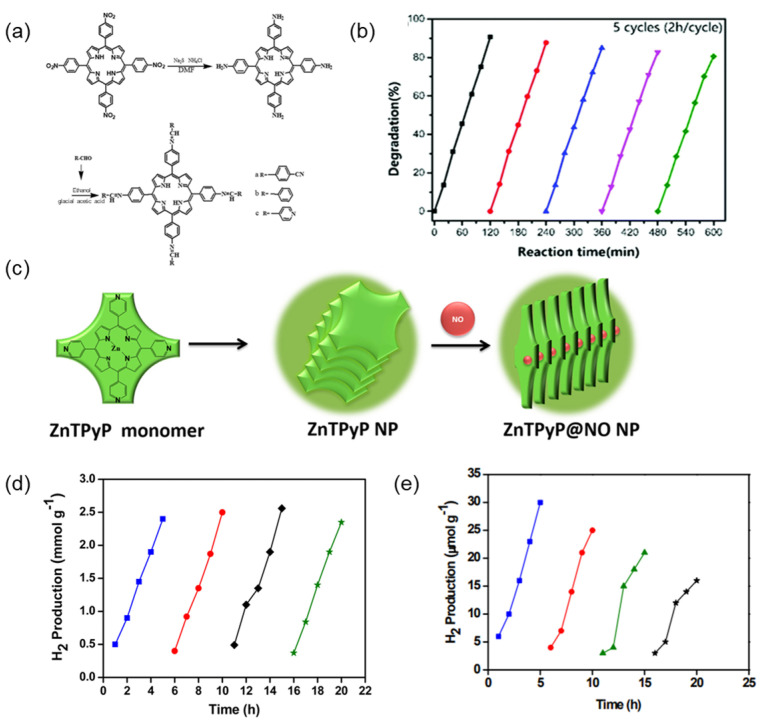
(**a**) Synthetic routes of TCyPPP, TbePPP, and TPyPPP; (**b**) Stability test of self-assembled photocatalytic activity of TCyPPP [129]; (**c**) Schematic diagram of the synthesis of self-assembled ZnTPyP nanoparticles and ZnTPyP@NO nanoparticles [130]; (**d**) Photocatalytic cycle of SA-PtPFTPP; (**e**) Photocatalytic cycle of SA-PtTPP [131].

Chemical modification of peripheral groups or alteration of molecular interactions, such as π-π interactions, hydrogen bonding, hydrophobic interactions, coordination ligands, etc., is an effective strategy to improve the stability of porphyrin assemblies [126,127,128]. The porphyrin molecule has a conjugated macrocyclic structure. The π-π interaction is one of the main driving forces for its self-assembly, and improving the π-π interaction between porphyrin molecules makes the porphyrin assembly more stable in solution. Hydrogen bonding is a strong intermolecular interaction, and the stability of porphyrin assemblies can be significantly improved by introducing hydrogen bonding donors or acceptors into porphyrin molecules. For example, Pei et al. [129] prepared three porphyrin derivatives, TCyPPP, TbePPP, and TPyPPP, from tetra-(4-aminophenyl) porphyrin (Figure 13a). The prepared porphyrin molecules can self-assemble into nanostructures by intermolecular interactions. The self-assemblies showed more efficient catalytic activity in the degradation of rhodamine B and higher cyclability compared to their free monomers (Figure 13b). Wang et al. [130] prepared stable nano-ZnTPyP@NO particles via coordinating nitric oxide (NO) with the core Zn ions of zinc meso-tetrad (4-pyridyl) porphyrin (ZnTPyP) and subsequent self-assembling (Figure 13c). The self-assembled particles were chemically stable and showed no loss after 180 days of storage at room temperature.

Compared to π-π stacking, F-F interactions generally provide higher chemical and thermal stability. Fluorine atoms exhibit unique characteristics, such as low polarization and high ionic energy. Structures stabilized by F-F interactions are less susceptible to chemical or thermal loads. To evaluate the photostability of SA-PtPFTPP and SA-PtTPP, Bodedla et al. [131] prepared SA-PtPFTPP and SA-PtTPP supramolecules via self-assembling of fluorinated porphyrin and unmodified porphyrin monomers, respectively. Consecutive cycles of photocatalytic hydrogen evolution showed that SA-PtPFTPP exhibited constant hydrogen production while SA-PtTPP significantly decreased with the cycle times (Figure 13d,e), indicating the self-assemblies composed of fluorinated porphyrin molecules had higher stability and reusability.

### 4.2. Core Metallization

The porphyrin-based metal complexes in nature usually have identical structures that promote the formation of ordered assembly arrays. The metal centers endow them with versatile functions. In heterogeneous porphyrin systems, the central metal of metalloporphyrin is considered to be one of the critical factors determining its stability and activity [132,133,134]. The coordination interaction between metal ions and nitrogen atoms of porphyrin rings is essential in porphyrin chemistry. This coordination interaction involves the formation of coordination bonds between metal ions and the core nitrogen atoms and stable metalloporphyrin complexes. Change of the central metals may result in significant differences in the electronic structure, axial bonding/interaction, and geometry of the periphery substitution [135].

**Figure 14:**
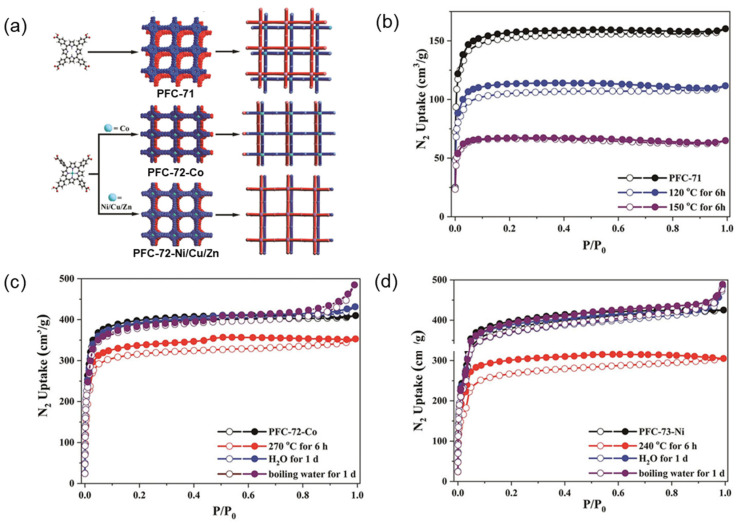
(**a**) Schematic structures of PFC-71, PFC-72-Co, and PFC-73-Ni/Cu/Zn; (**b**) N_2_ adsorption isotherm (77 K) of PFC-71 after different treatments; (**c**) N_2_ adsorption isotherm of PFC-72 after different treatments; (**d**) N_2_ adsorption isotherm of PFC-73 after different treatments [136].

Higher electronegativity means a stronger attraction to electrons, and substituted metal ions for hydrogen ions at the porphyrin center will bring a more significant electronegativity difference in the macrocyclic skeleton, resulting in increased polarizability and electron cloud distortion. Yin et al. [136] began with a four-bit carboxylate porphyrin ligand and continuously adjusted the synthesis conditions to obtain a topically guided hydrogen-bonded organic framework (PFC-71). Metallized porphyrin produced the same network but with more significant orbital overlap between the interlayer of porphyrin cores (PFC-72 and PFC-73) (Figure 14a). Treatment of PFC-71 with water at 120 °C and 150 °C for 6 h resulted in significant structural collapse (Figure 14b), while the metallized analogues exhibited significantly improved robustness (Figure 14c,d).

### 4.3. Arrangement of Internal Spaces

Some porphyrin molecules can adjust the delocalization of the internal spatial arrangement of excited electrons and prolong the lifetime of charges through non-covalent interactions during self-assembly, thereby improving the efficiency and activity of photocatalysis. Moreover, they can form a relatively stable, rigid structure in self-assembly to ensure excellent stability [60,130,137].

**Figure 15:**
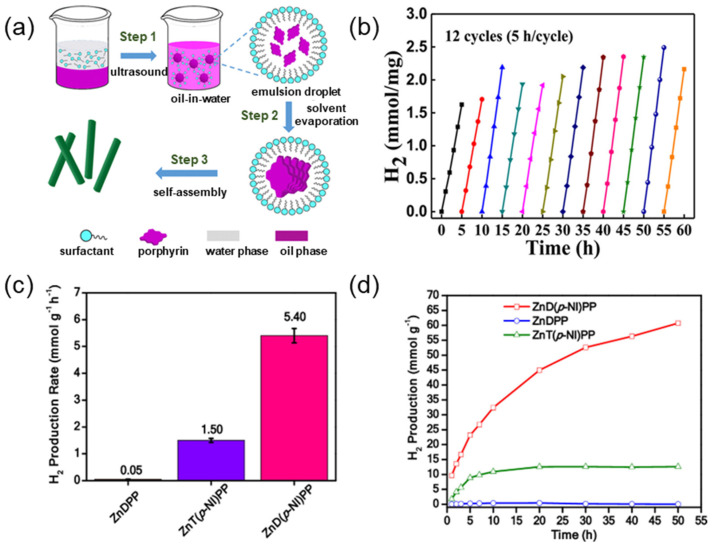
(**a**) Schematic diagram of the formation of self-assembled InTPP nanostructures by an emulsion-based self-assembly process; (**b**) Photocatalytic recovery experiments of InTPP [137]; (**c**) Photocatalytic rates of different photocatalysts at 5 h; (**d**) Hydrogen production plots over different photocatalysts under light irradiation [138].

Liu et al. [137] enabled In (III) racemic tetraphenyl porphyrin chloride (InTPP) in microemulsions to assist nucleation and growth through non-covalent interactions (Figure 15a). A series of uniform nanorods with controllable aspect ratios were fabricated by adjusting the conditions, including the concentration of the surfactant concentration. The orderly molecular packing structure allows for effective light absorption and stability of the resulting porphyrin nanocrystals under visible light. These nanocrystals exhibit excellent photocatalytic hydrogen production activity and photostability compared to commercially available InTPP porphyrin powders (Figure 15b). Boddela et al. [138] prepared nanowires with definite morphology via the self-assembly of ZnD(p-NI)PP. ZnD(p-NI)PP exhibited excellent photocatalytic performance and stability compared with ZnT(p-NI)PP and ZnDPP (Figure 15c,d).

## 5. Photocatalytic Application of Porphyrin Supramolecules

### 5.1. Hydrogen Evolution from Water

Various porphyrin-based artificial photosystems, including molecule-based, porphyrin metal-organic frameworks, and porphyrin-based hybrid composites, have been constructed and used for solar-driven hydrogen production from water splitting [19,139,140,141,142,143,144]. Self-assembled porphyrin nanostructures have electronic coupling between porphyrin molecules due to their anisotropic arrangements, which enhances the separation and transfer efficiency of electrons and holes. The porphyrin supramolecules also exhibit unique composition, morphology, and size-dependent photocatalytic activity in hydrogen evolution [57,145,146,147].

**Figure 16:**
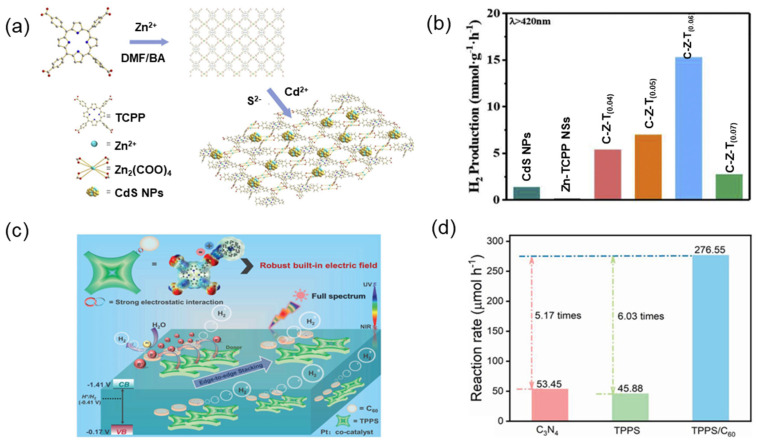
(**a**) Schematic diagram of the synthesis of C-Z-T nanocomposites; (**b**) Hydrogen production rates of CdS Nps and composites under visible light [149]; (**c**) Mechanism diagram of D-A supramolecular photocatalyst; (**d**) Hydrogen production rate at the full spectrum of TPPS/C60 [151].

The efficient photocatalytic activity in hydrogen production of supramolecular porphyrins can be achieved by adjusting their band structure and the separation ability of photogenerated carriers by metal ions [148]. A novel composite photocatalyst (C-Z-T) with porphyrin organic skeleton was constructed by in situ growing CdS nanoparticles on the surface of two-dimensional (2D) zinc porphyrin nanosheets (Zn-TCPP NSs) (Figure 16a) [149]. The photocatalyst showed excellent light absorption ability, and their heterostructure can effectively suppress the fast recombination of photogenerated electron-hole pairs. The photocatalytic hydrogen evolution rate can reach 15.3 mmol g^−1^ h^−1^ (Figure 16b).

As photocatalysts, the performance of porphyrin assemblies is limited by the low efficiency of charge separation. Construction of photosensitive composite materials is expected to alleviate this problem [150]. Yang et al. [151] constructed a TPPS/C60 hybrid photocatalyst with D-A structures by electrostatic interaction and π-π interaction. The D-A structure in the photocatalysts effectively promotes the transfer of electrons from porphyrin aggregates to C60 and significantly improves the separation efficiency of photogenerated charges. The results of photocatalytic production of hydrogen showed that TPPS/C60 had extremely high efficiency, where the catalytic activity was 6.03 times that of the TPPS aggregate alone (Figure 16d).

### 5.2. Reduction in CO_2_

The efficient utilization of solar energy for the photocatalytic reduction in carbon dioxide can solve the problems of energy shortage and global warming. Porphyrins and their self-assemblies can catalyze the reduction in carbon dioxide to value-added molecules such as methane and methanol under light irradiation due to excellent electronic and optical properties, light absorption capacity, and efficient electron transfer characteristics [152,153,154,155,156]. The selectivity and efficiency of CO_2_ reduction can be improved through the rational design of the supramolecular structure and reaction conditions. This also provides a new approach to reducing greenhouse gas emissions and realizing carbon recycling.

**Figure 17:**
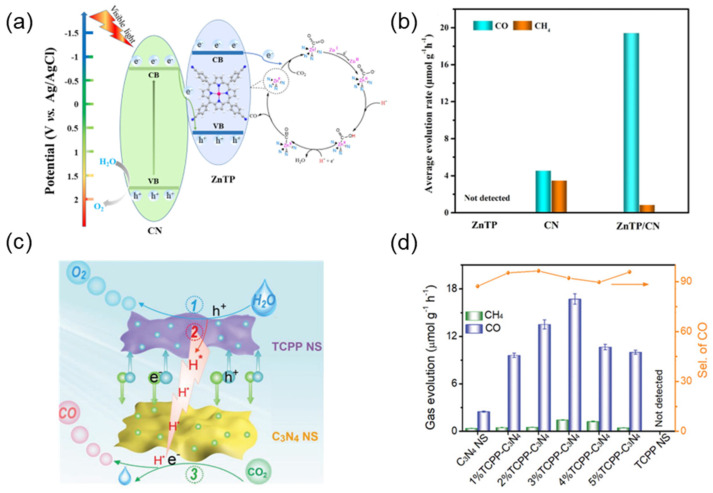
(**a**) Photoreduction mechanism of ZnTP/CN photocatalyst under illumination; (**b**) Natural gas product generation rates for ZnTP, CN, and ZnTP/CN [157]; (**c**) TCPP-C_3_N_4_ reaction mechanism diagram; (**d**) Visible light-driven CO_2_ photoreduction performance [159].

Jia et al. [157] designed and synthesized zinc (II) tetra-(4-cyanophenyl) porphyrin (ZnTP) assemblies and then anchored them to porous carbon nitride (C_3_N_4_) nanosheets (Figure 17a). The introduction of ZnTP nanoparticles expanded their visible light absorption range, and the creation of ZnTP/CN heterostructures in the hybrids endows them with significantly enhanced activity in CO_2_ photoreduction. CH_4_ and CO are reduction products. After the reaction, ZnTP did not produce significant carbonaceous products. The original CN nanosheets showed that CO and CH_4_ formation rates were 4.5 and 3.4 μmol g^−1^ h^−1^, and the CO selectivity was 57.4%. The CO yield of ZnTP/CN composites is significantly increased to 19.4 μmol g^−1^ h^−1^, which is about 4.3 times that of CN nanosheets alone, and the CO selectivity is 95.8%. (Figure 17b). In addition, the formation of S-type heterojunctions improved the separation and transfer efficiency of photogenerated carriers [158]. Chen et al. [159] designed a heterojunction between C_3_N_4_ and supramolecular porphyrin nanosheets (NSs) (Figure 17c). The S-type heterojunctions performed well in visible-light-driven CO_2_ reduction, in which the maximum CO production rate was about 6.8 times that of the original C_3_N_4_ (Figure 17d).

### 5.3. Generation of Coenzyme NADH

The generation of nicotinamide adenine dinucleotide (NADH) from its oxidized congener (NAD^+^) is crucial in cellular metabolism, especially in energy production and redox balance. At present, many research studies about photocatalytic systems focused on the sustainable cycling of NAD^+^/NADH and improving the efficiency and activity of NADH regeneration [11,160,161,162,163]. Applying porphyrin supramolecules in the regeneration of coenzyme NADH reflects its vital role in biocatalysis and organic synthesis [164].

The porphyrin ring, the key component of light harvesting and electron transport, reduces NAD^+^ to NADH by capturing two electrons and one proton under light irradiation [165,166]. This process not only mimics the natural photosynthesis but also provides essential coenzymes for various NADH-dependent enzymatic reactions. The design and construction of stable and excellent photocatalytic systems for NADH regeneration also play a crucial role in artificial photosynthesis. Researchers are committed to the design and construction of photocatalysts with excellent photocatalytic reduction capabilities. In the past few decades, porphyrin self-assembled nanostructures that mimic chlorophyll have been applied in NADH regeneration [167,168].

**Figure 18:**
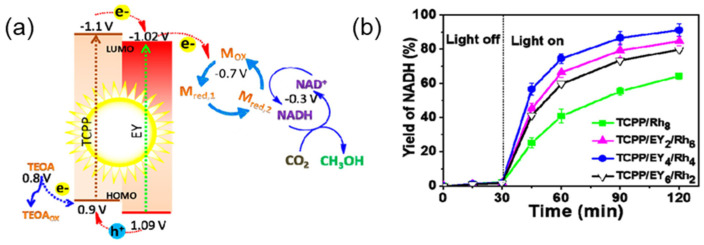
(**a**) Schematic diagram of energy levels and electron transfer in a biomimetic artificial photosynthesis system; (**b**) Time curves for photocatalytic regeneration of NADH by NAD with different supramolecular assemblies [169].

Ji et al. [169] designed and synthesized a TCPP/EYx/Rh_8−x_ macromolecule from porphyrin (TCPP), eosin Y (EYx), and electronic medium M (Rh_8−x_). The macromolecule self-assembled into a supramolecular assembly that mimics the chloroplasts through non-covalent interactions. The supramolecular assembly improved the yield of NADH from 15% of free macromolecules to 91%, and the yield of methanol was approximately 12-fold when the NADH regeneration system was coupled with dehydrogenase (Figure 18b). The research represents a milestone in achieving efficient solar energy conversion and CO_2_ reduction in a clean and sustainable way.

### 5.4. Removal of Organic Pollutants

In recent years, various organic pollutants were continuously discharged into the water environment, accompanied by the continuous development of industrialization. These pollutants attracted significant attention because of their potent toxicity, high carcinogenicity, and non-degradability [170,171]. Porphyrin supramolecules have great flexibility and high efficiency in the photocatalytic degradation of pollutants due to the tunability of the structure and wide-spectrum light absorption [172,173,174]. Photocatalytic mechanism studies have shown that porphyrin supramolecules can generate efficient electron-hole pairs under visible light irradiation and quickly separate to generate reactive oxygen species with strong oxidizing properties (such as hydroxyl radicals, superoxide anions, etc.), so as to achieve effective degradation of different organic pollutants [175,176]. In practical applications, porphyrin supramolecular materials exhibit a wide range of contaminant removal capabilities. For example, in dye wastewater treatment, the degradation efficiency of azo dyes and fluorescent dyes is significantly higher than that of traditional photocatalysts. In addition, the material also exhibits excellent performance in degrading pesticides, pharmaceutical contaminants, and phenolic compounds.

**Figure 19:**
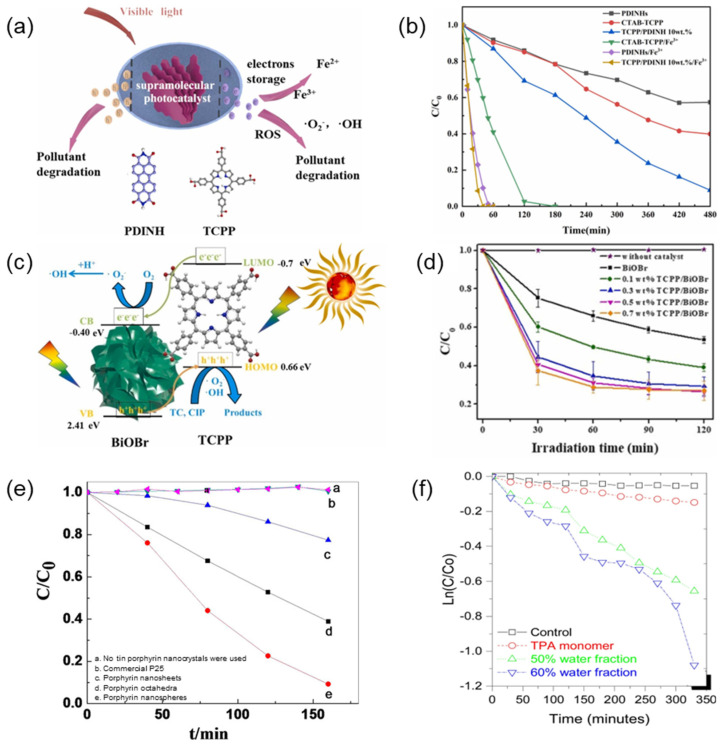
(**a**) Schematic diagram of TCPP/PDINH photocatalytic system; (**b**) Degradation rate of phenol with different photocatalysts under visible light [180]; (**c**) Schematic diagram of TCPP/BiOBr photocatalytic system; (**d**) Degradation rate of tetracycline with different photocatalysts under visible light [182]; (**e**) Degradation rates of methyl oranges under visible light by different photocatalysts [184]; (**f**) Degradation rate of rhodamine B under visible light by different photocatalysts [186].

Firstly, phenol, as a common industrial pollutant, poses a serious threat to the environment due to its toxicity and refractory degradability. Under visible light irradiation, porphyrin supramolecular materials can effectively excite and generate electron-hole pairs and quickly separate, and the generated reactive oxygen species can efficiently oxidize phenol molecules, destroy their structure, and finally convert them into harmless small molecules [171,177,178,179]. Lu et al. [180] constructed supramolecular photocatalysts for porphyrin (TCPP) and perylene diimide (PDINH) (Figure 19a) and enhanced the photocatalytic activity of organic pollutants such as phenol by using Fe (III) ions to promote interfacial charge transfer (IFCT). The degradation rate of phenol by TCPP/PDINH/Fe^3+^ reached 100% within 40 min (Figure 19b).

Secondly, tetracycline, as a widely used antibiotic, poses a serious threat to the environment and human health. Porphyrin supramolecular materials can rapidly degrade tetracycline through their strong light absorption capacity and efficient charge separation [181]. Under visible light irradiation, the degradation rate of tetracycline significantly increased, showing the great potential of the material in antibiotic wastewater treatment. Xing et al. [182] prepared TCPP/BiOBr composites by integrating TCPP self-assemblies with bismuth hydrobromide (BiOBr) (Figure 19c). Compared with pure BiOBr, the composites exhibited remarkably improved photocatalytic activity in tetracycline degradation attributed to their enhanced light absorption by the introduction of TCPP (Figure 19d). At the same time, the suitable interfaces between the TCPP self-assemblies and BiOBr improved the charge separation efficiency and promoted the formation of active species.

In addition, as a common azo dye, the wastewater treatment of methyl orange has always been a difficult problem in the field of environmental protection. Through the synergistic effect of photogenerated electrons and holes, the mineralization rate of methyl orange was significantly improved [183]. Zhong et al. [184] used tin (IV) racemic tetraphenyl porphyrin dichloride (tin porphyrin) to synthesize hierarchical active nanocrystals (nanocrystals, octahedrons, and nanospheres) to degrade methyl orange under visible light irradiation and found that tin porphyrin showed excellent photocatalytic degradation of methyl orange compared with the photocatalysts without tin porphyrin. Among them, the degradation efficiency of tin porphyrin nanospheres can reach up to 90% (Figure 19e).

Finally, as a fluorescent dye, rhodamine B has environmental persistence and biological toxicity that cannot be ignored. Porphyrin supramolecular materials also exhibit excellent performance in the process of photocatalytic degradation of rhodamine B [185]. Through the synergistic effect of photogenerated electrons and holes, the fluorescence intensity of rhodamine B is significantly reduced, effectively destroying its molecular structure. D. Duong La et al. [186] synthesized a new porphyrin-based nanomaterial using tetrakis-triphenylamine porphyrin (TTPAP) by the solvent-phobic self-assembly method. Under simulated visible light irradiation, both porphyrin microstructures (the bar water content is 50% and the strip water content is 60%) showed reasonable photocatalytic activity for Rhodamine B in aqueous media, and the banded porphyrin aggregates showed better performance than the rod-like aggregates (Figure 19f).

## 6. Conclusions

This paper systematically reviewed the advancement of porphyrin supramolecules in molecular design, construction approach, mechanism of charge separation and transfer, photocatalytic stability, application in hydrogen evolution, carbon dioxide reduction, regeneration of NADH, and removal of organic pollutants. Different porphyrin supramolecular structures can be obtained through self-assembly, co-assembly, and polymer-induced assembly. The introduction of heterojunctions, donor-acceptor (D-A) structures, and cocatalysts is conducive to charge separation and transport efficiency. Chemical modification, core metallization, and adjustment of the internal spatial arrangement of porphyrin help stabilize the supramolecular structures. Moreover, porphyrin supramolecules exhibit great potential in energy conversion and environmental governance due to their tailable structures and flexible light responsive properties.

However, there are some challenges that limit the development of porphyrin supramolecular photocatalysts. Firstly, precise control of the structure and performance of the porphyrin assemblies is challenged. Secondly, there are bottlenecks in improving charge separation and transport efficiency, especially in complex reaction systems. Thirdly, the stability of porphyrin self-assemblies needs to be further improved, although the problem of porphyrin molecules being easily dissolved by solution is solved by supramolecular self-assembly. Finally, now most porphyrin-based supramolecular assemblies are prepared and investigated in the laboratory, their large-scale preparation and practical application in industry is still a huge challenge. Additionally, future research should focus on the following aspects: (1) The development of novel, low-cost, noble-metal-free porphyrin self-assemblies is particularly important for future commercial applications. (2) The photocatalytic performance of porphyrins and their derivatives needs to be further optimized, especially the generation and separation efficiency of carriers. (3) Integration with other functional units to achieve synergistic effects. (4) Facile preparation, stability, and reusability of the photocatalysts should be paid more attention to use them in practical applications. These studies will broaden the application of porphyrin supramolecules in photocatalysis.

## Data Availability

No applicable.
